# Experimental quantum secure network with digital signatures and encryption

**DOI:** 10.1093/nsr/nwac228

**Published:** 2022-10-22

**Authors:** Hua-Lei Yin, Yao Fu, Chen-Long Li, Chen-Xun Weng, Bing-Hong Li, Jie Gu, Yu-Shuo Lu, Shan Huang, Zeng-Bing Chen

**Affiliations:** National Laboratory of Solid State Microstructures, School of Physics, Collaborative Innovation Center of Advanced Microstructures, Nanjing University, Nanjing 210093, China; MatricTime Digital Technology Co. Ltd., Nanjing 211899, China; National Laboratory of Solid State Microstructures, School of Physics, Collaborative Innovation Center of Advanced Microstructures, Nanjing University, Nanjing 210093, China; National Laboratory of Solid State Microstructures, School of Physics, Collaborative Innovation Center of Advanced Microstructures, Nanjing University, Nanjing 210093, China; National Laboratory of Solid State Microstructures, School of Physics, Collaborative Innovation Center of Advanced Microstructures, Nanjing University, Nanjing 210093, China; National Laboratory of Solid State Microstructures, School of Physics, Collaborative Innovation Center of Advanced Microstructures, Nanjing University, Nanjing 210093, China; National Laboratory of Solid State Microstructures, School of Physics, Collaborative Innovation Center of Advanced Microstructures, Nanjing University, Nanjing 210093, China; National Laboratory of Solid State Microstructures, School of Physics, Collaborative Innovation Center of Advanced Microstructures, Nanjing University, Nanjing 210093, China; National Laboratory of Solid State Microstructures, School of Physics, Collaborative Innovation Center of Advanced Microstructures, Nanjing University, Nanjing 210093, China; MatricTime Digital Technology Co. Ltd., Nanjing 211899, China

**Keywords:** quantum digital signature, information-theoretical security, secret sharing, one-time universal_2_ hashing, cryptography toolbox

## Abstract

Cryptography promises four information security objectives, namely, confidentiality, integrity, authenticity and non-repudiation, to support trillions of transactions annually in the digital economy. Efficient digital signatures, ensuring integrity, authenticity and non-repudiation of data with information-theoretical security are highly urgent and intractable open problems in cryptography. Here, we propose a high-efficiency quantum digital signature (QDS) protocol using asymmetric quantum keys acquired via secret sharing, one-time universal_2_ hashing and a one-time pad. We just need to use a 384-bit key to sign documents of lengths up to 2^64^ with a security bound of 10^−19^. If a one-megabit document is signed, the signature efficiency is improved by more than 10^8^ times compared with previous QDS protocols. Furthermore, we build the first all-in-one quantum secure network integrating information-theoretically secure communication, digital signatures, secret sharing and conference key agreement and experimentally demonstrate this signature efficiency advantage. Our work completes the cryptography toolbox of the four information security objectives.

## Teaser

A new paradigm of quantum digital signatures is proposed and experimentally demonstrated, improving the efficiency by hundreds of millions of times and making it as practical as quantum secure communication.

## INTRODUCTION

Fast developing driverless, blockchain and artificial intelligence technologies, as well as digital currency systems, will soon require a more robust network with security against quantum attacks [1]. A promising blueprint for such a network ensures hash functions, encryption algorithms and digital signatures with information-theoretical security, which cannot be met in the current internet with public-key infrastructure [2]. Currently, widely implemented one-way hash functions, such as Message Digest 5 [3] and Secure Hash Algorithm 1 [4], are no longer secure. For example, since 2017, one can utilize two different files to obtain the identical hash value after conducting Secure Hash Algorithm 1 [5]. Additionally, in 2020, the public-key encryption algorithms [6–8], based on the computational complexity of factorization and the discrete logarithm, have both been compromised at the 795-bit level [8]. More seriously, quantum computers can in principle attack public-key cryptosystems with any number of bits [9].

Unlike public-key cryptography, one-time pad (OTP) encryption based on a symmetric key allows a message to be transmitted with information-theoretical confidentiality [10] over a standard communication channel, with the symmetric key being securely established using quantum key distribution [11] and the attackers’ computational power being unrestricted. Currently, there are several experimental demonstrations and commercial applications of quantum key distribution around the world [12–16].

**Figure 1.**
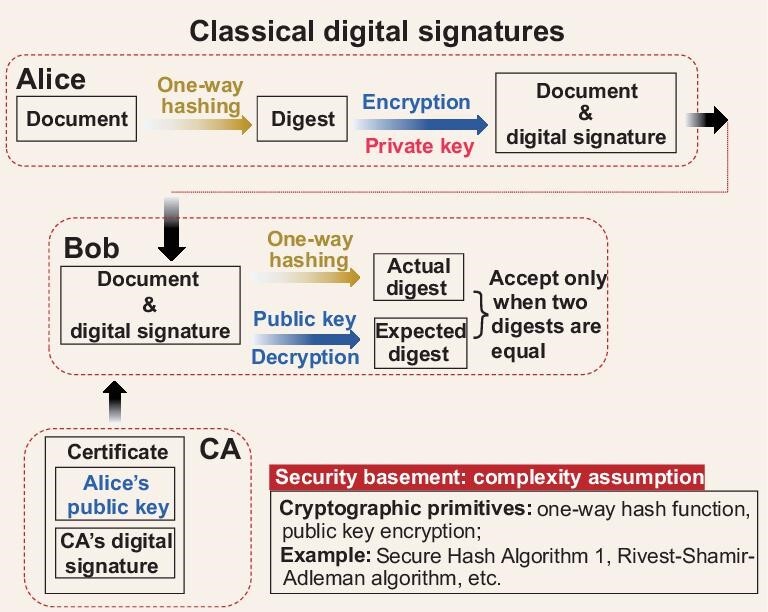
Schematic diagram of classical digital signatures. Alice uses a private key to encrypt the digest to obtain the signature, where the digest is acquired via a fixed one-way hash function. She sends the document along with the signature to Bob. Bob utilizes the same one-way hash function and the corresponding public key to acquire two digests. Then, he only accepts the signature if the two digests are identical. A digital certificate issued by the certificate authority (CA) guarantees the validity of the public key. Here, we omit the generation process of the private and public keys.

Quantum key distribution [11–16] and quantum secure direct communication [17,18] only ensure confidentiality, which is, however, an incomplete solution to the remaining cryptographic tasks. Three other fundamental information security objectives are integrity, authenticity and non-repudiation [2]. These other tasks are usually realized in classical cryptosystems by digital signatures using one-way hash functions and public-key encryption algorithms, as shown in Fig. 1. Digital signatures [2] play a vital role in software distribution, emails, web browsing and financial transactions, but they become insecure as one-way hash functions and public-key encryption algorithms used therein are breakable by either classical or quantum computers [19].

Unlike classical solutions [2], quantum digital signatures (QDSs) use quantum laws to sign a document with information-theoretical integrity, authenticity and non-repudiation. In 2001, the first rudiment of the QDS was introduced [20], but it could not be implemented. Developments in the last decade have removed impractical requirements of the QDS, such as a high-dimensional single-photon state, quantum memory [21] and secure quantum channels [22–26], enabling demonstrations of the QDS in various experimental systems [27–31]. However, the resulting schemes still have serious limitations that require an approximately 10^5^-bit key to sign only a one-bit document. For a gigahertz system, the best signature rate reported thus far is less than 1 time per second (tps) for one bit at a 100-km transmission distance [30]. Additionally, it is unknown how to efficiently sign multi-bit documents with information-theoretical security [32], which makes all known single-bit-type QDS protocols far from practical applications [20–25,27–31]. Thus, a high-efficiency QDS that is as feasible as quantum private communication (using quantum key distribution) [12] is highly desirable and remains an unsolved open problem. Note that a probabilistic one-time delegation of the signature authority protocol was proposed and demonstrated using entanglement correlation [33].

Here, we propose a one-time universal_2_ hashing (OTUH)-QDS protocol capable of signing an arbitrarily long document with information-theoretical security. For example, just with a 384-bit key, our protocol can sign documents of lengths up to 2^64^ with a security bound of 10^−19^. Furthermore, we propose, for the first time, the concept of OTUH: a completely random and different universal_2_ hash function [34] used for each digital signature. Our protocol not only uses OTUH and OTP as the underlying cryptography layer, but also uses secret sharing to realize the perfect bits correlation of the three parties, and then builds an asymmetric key relationship for Alice and Bob. Secret sharing can be implemented with information-theoretical security using quantum secret sharing, quantum key distribution or future quantum internet with solid-state entanglement. Additionally, we simulate the performances of our OTUH-QDS protocol based on various quantum communication protocols. The simulation results show that, for a gigahertz system, the signature rates are more than 10^4^ tps in the metropolitan area, which represents an efficiency improvement of at least eight orders of magnitude for signing a one-megabit document. Additionally, we experimentally construct a quantum secure network to realistically demonstrate cryptographic primitives [2] with information-theoretical security, such as private communication, digital signatures, secret sharing and conference key agreement. In our experiment, the signature efficiency can be achieved 1.43 × 10^8^ times that of the previous work in Ref. [23] considering the improvements in signature rates for signing a 130 250-byte document over a 101-km fiber, and the security bound is as small as 10^−32^, which shows a significant advantage.

## RESULTS

### Efficient QDS protocol

**Figure 2.**
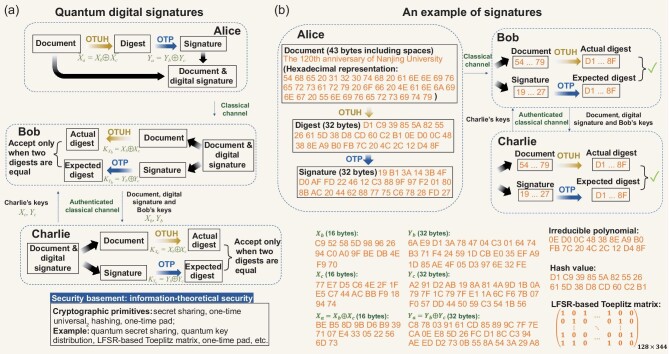
Schematic diagram for the QDS and the corresponding example. (a) Compared with the classical scheme, Charlie plays the role of a certificate authority. Alice’s key can be viewed as a quantum private key, while Bob’s key is a quantum public key; in our protocol, they are asymmetric. The information-theoretically secure OTUH replaces the fixed one-way hash function. Here, we omit the pre-distribution stage for information-theoretically secure asymmetric quantum key generation, which replaces the classical private and public key generation process. (b) As an example, we sign a document ‘The 120th anniversary of Nanjing University.’ The details of the document, digest, signature, irreducible polynomial and key bit strings are shown in hexadecimal format.

Before executing our OTUH-QDS protocol, three parties, Alice, Bob and Charlie, will perform the pre-distribution stage, which is analogous to the private and public key generation procedure in classical digital signatures. Alice, Bob and Charlie each have two key bit strings {*X_a_*, *X_b_*, *X_c_*} with *n* bits and {*Y_a_*, *Y_b_*, *Y_c_*} with 2*n* bits, where the key bit strings meet the perfect correlations *X_a_* = *X_b_* ⊕ *X_c_* and *Y_a_* = *Y_b_* ⊕ *Y_c_*, respectively. The pre-distribution stage can be realized using quantum communication protocols (see the Methods section), such as quantum key distribution [35–39] and quantum secret sharing [40–44]. Before executing the signature, Alice is the signer, and both Bob and Charlie can be the receiver because of the symmetry between Bob and Charlie. Here, we suppose that Alice signs an *m*-bit document, denoted by *Doc*, to the desired recipient, Bob. Therefore, Bob is the specified receiver, and Charlie automatically becomes the verifier. Our proposed approach utilizes secret sharing, OTUH and OTP to generate and verify signatures, as shown in Fig. 2(a). We remark that the keys of signer, Alice, and receiver, Bob, are asymmetric because *X_a_* ≠ *X_b_* and *Y_a_* ≠ *Y_b_*. After completing the pre-distribution stage, the three parties can implement the signature stage at any time.

(i) *Signing of Alice.*—First, Alice uses a local quantum random number, which can be characterized by an *n*-bit string *p_a_* (see the online supplementary material), to randomly generate an irreducible polynomial *p*(*x*) of degree *n* [2]. Second, she uses the initial vector (key bit string *X_a_*) and irreducible polynomial (quantum random number *p_a_*) to generate a random linear feedback shift register-based (LFSR-based) Toeplitz matrix [45] *H_nm_*, with *n* rows and *m* columns. Third, she uses a hash operation with *Hash* = *H_nm_* · *Doc* to acquire an *n*-bit hash value of the *m*-bit document. Fourth, she exploits the hash value and the irreducible polynomial to constitute the 2*n*-bit digest *Dig* = (*Hash*||*p_a_*). Fifth, she encrypts the digest with her key bit string *Y_a_* to obtain the 2*n*-bit signature *Sig* = *Dig* ⊕ *Y_a_* using OTP. Finally, she uses the public channel to send the signature and document {*Sig, Doc*} to Bob.

(ii) *Verification of Bob.*—Bob uses the authentication classical channel to transmit the received {*Sig, Doc*}, as well as his key bit strings {*X_b_*, *Y_b_*}, to Charlie. Then, Charlie uses the same authentication channel to forward his key bit strings {*X_c_*, *Y_c_*} to Bob. Bob obtains two new key bit strings $\lbrace K_{X_{b}}=X_{b}\oplus X_{c},\, K_{Y_{b}}=Y_{b}\oplus Y_{c}\rbrace$ by the XOR operation. Bob exploits $K_{Y_{b}}$ to obtain an expected digest and bit string *p_b_* via XOR decryption. Bob utilizes the initial vector $K_{X_{b}}$ and irreducible polynomial *p_b_* to establish an LFSR-based Toeplitz matrix. Bob uses a hash operation to acquire an *n*-bit hash value and then constitutes a 2*n*-bit actual digest. Bob will accept the signature if the actual digest is equal to the expected digest. Then, he informs Charlie of the result. Otherwise, Bob rejects the signature and announces aborting the protocol.

(iii) *Verification of Charlie.*—If Bob announces that he accepts the signature, Charlie then uses his original key and the key sent to Bob to create two new key bit strings $\lbrace K_{X_{c}}=X_{b}\oplus X_{c},\, K_{Y_{c}}=Y_{b}\oplus Y_{c}\rbrace$. Charlie employs $K_{Y_{c}}$ to acquire an expected digest and bit string *p_c_* via XOR decryption. Charlie uses a hash operation to obtain an *n*-bit hash value and then constitutes a 2*n*-bit actual digest, where the hash function is an LFSR-based Toeplitz matrix generated by initial vector $K_{X_{c}}$ and irreducible polynomial *p_c_*. Charlie accepts the signature if the two digests are identical. Otherwise, Charlie rejects the signature.

To show more clearly how our protocol works, Fig. 2(b) shows an example of signing the document ‘The 120th anniversary of Nanjing University.’

### Security proof

In a QDS scheme, either Alice or Bob can be the attacker. Thus, Alice and Bob distrust each other, whereas the verifier, Charlie, is always trusted. Bob and Charlie will cooperate to counter Alice’s repudiation attack. Alice and Charlie will collaborate to counter Bob’s forgery attack. Besides, we also consider the robustness of our protocol.

#### Security against forgery

When Charlie accepts the tampered document forwarded by Bob, Bob’s forgery attack is considered successful. There are two cases of Bob’s forgery attack. First, Bob can generate a new document and signature if Alice has not signed a document at all. Second, Bob can change the document and signature if Alice has signed the document. According to our protocol, Charlie accepts the signature if and only if he obtains the identical digest by decrypting the signature with OTP and hashing the document with OTUH, respectively. Note that before Bob forwards the document, signature, and his key bit strings to Charlie, Bob cannot obtain the key bit strings of Charlie. In the first case, Bob has no information since Alice did not send any information. The only thing Bob can do is correctly guess Alice’s key bit strings *X_a_* and *Y_a_*, i.e. guessing Charlie’s key bit strings *X_c_* and *Y_c_* based on *X_a_* = *X_b_* ⊕ *X_c_* and *Y_a_* = *Y_b_* ⊕ *Y_c_*. The probability of guessing correctly is at most 1/2^*n*^ since Bob has no information of key bit strings *X_a_* and *Y_a_* with *n* and 2*n* bits, respectively. In the second case, Bob also has no information on the universal_2_ hash function (initial vector *X_a_* and irreducible polynomial *p_a_* for the LFSR-based Toeplitz matrix [45]) used by Alice since the digest has been encrypted to a signature using OTP. Besides, Bob cannot obtain any information from the previous signing round because their keys are refreshed and the corresponding universal_2_ hash function is updated in each round in our protocol. Compared to guessing the key bit strings of Alice or Charlie, Bob’s best strategy is to guess the irreducible polynomial *p_a_* of the LFSR-based Toeplitz matrix. The collision probability of universal_2_ hashing by the LFSR-based Toeplitz matrix can be determined by [45] *m*/2^*n* − 1^ (see the Methods section), which implies that one can find two distinct documents with identical hash values by randomly guessing the irreducible polynomial *p_a_*. Therefore, for any case, the probability of a successful forgery can be bounded by


(1) \begin{equation*} \epsilon _{\rm for}=\frac{m}{2^{n-1}}, \end{equation*}

which is identical to the collision probability given in [45]. Here *m* is the length of the document *Doc* and *n* is the order of the irreducible polynomial *p_a_*. We emphasize that the failure probability or guessing probability [46] of 3*n* bits of quantum key distribution is extremely small, though they are non-zero. For example, the guessing probability is smaller than 10^−3277^ [46] given a failure probability bounded by 10^−9^.

Note that our proof is information-theoretically secure, even though Bob has unlimited computing power. We emphasize the importance of our proposed OTUH, where the universal_2_ hash function is only used once and then updated. Bob cannot obtain any information from the previously signed round because their keys and irreducible polynomial are refreshed in every round. Bob cannot do anything at all apart from randomly guess. Moreover, before Bob sends the document and signature to Charlie, Bob cannot be sure if he guessed correctly even if he exhausts all the possibilities. Bob’s forgery attack in our OTUH-QDS protocol is successfully related to Eve’s attack in information-theoretically secure message authentication [45,47] (details can be found in the online supplementary material).

#### Security against repudiation

Successful repudiation means that Alice makes Bob accept the signature, while Charlie rejects it. For Alice’s repudiation attacks, Bob and Charlie are both honest and trust each other. Note that Bob and Charlie must forward their key bit strings to each other through an authenticated classical channel. The authenticated channel used ensures that Alice knows about the transmitted information between Bob and Charlie but cannot tamper with it. Then, Bob and Charlie can recover the identical key bit strings through the XOR operation $K_{X_{b}}=X_{b}\oplus X_{c}=K_{X_{c}}$ and $K_{Y_{b}}=Y_{b}\oplus Y_{c}=K_{Y_{c}}$. Bob and Charlie obtain the same irreducible polynomial *p_b_* = *p_c_* through OTP decryption. They will make the same decision for the same document, signature, key bit strings and irreducible polynomial. Therefore, our QDS protocol is naturally immune to repudiation. The probability of repudiation is zero when we ignore the insignificant failure probability of secure message authentication.

Note that in all known QDS protocols, the symmetry between Bob and Charlie is used to counter Alice’s repudiation attacks. Compared to partial symmetry in previous protocols, Bob and Charlie will have identical key bit strings in our protocol after performing the QDS process. In addition, there is no help for Alice’s repudiation attack, even though she is dishonest in the pre-distribution stage because we allow Alice to obtain all information from Bob (Charlie) about *X*_*b*(*c*)_ and *Y*_*b*(*c*)_.

#### Robustness

The robustness quantifies the probability that Bob rejects the signature when the three parties are truthful. If Alice, Bob and Charlie are all truthful, there are the relations of irreducible polynomial *p_a_* = *p_b_* = *p_c_* and key bit strings $X_a=K_{X_b}=K_{X_c}$ and $Y_a=K_{Y_b}=K_{Y_c}$. Thus, they will use the same universal_2_ hash function and generate the same actual digest. The signature will be accepted naturally. The probability of honest aborting is zero, though, in the pre-distribution stage, we ignore the insignificant failure probability of classical bit error correction of quantum communication protocols.

Note that the verification step of error correction in quantum key distribution and quantum secret sharing is usually realized using the universal_2_ hashing and OTP, which is related to the information-theoretically secure message authentication [47]. The verification step ensures that the classical bit error correction is successful with a small failure probability.

Thus, if one uses 128-bit and 256-bit keys for OTUH and OTP, respectively, the security bound of our OTUH-QDS protocol is less than 2^64^/(2^128 − 1^) ≈ 1.1 × 10^−19^ for documents of lengths up to 2^64^.

To sum up, the practicality of our QDS protocol is enabled by, first of all, asymmetric quantum cryptography, which provides asymmetric quantum keys, between the signer and the two receivers, acquired via secret sharing. Then, using asymmetric quantum keys in the OTP and OTUH, our QDS protocol is information-theoretically secure against forgery and repudiation. The main reasons for its signature efficiency advantage will be further discussed in our experimental implementation shown below.

### Simulation results of the QDS

Secret sharing in the pre-distribution stage allows the key bit strings of Alice, Bob and Charlie to satisfy *X_a_* = *X_b_* ⊕ *X_c_* and *Y_a_* = *Y_b_* ⊕ *Y_c_*, which can be implemented with information-theoretical security using quantum secret sharing, quantum key distribution or future quantum internet with solid-state entanglement. Meanwhile, a full-blown quantum internet, with functional quantum computers and quantum repeaters as nodes connected through quantum channels, is being developed. The first prototype of the quantum internet has been realized with remote solid-state qubits [48] in multi-party entanglements applicable to secret sharing. To date, there is no workable quantum-secure asymmetric cryptosystem. With the help of secret sharing, our framework represents the first practical quantum asymmetric cryptosystem (*X_a_* ≠ *X_b_* and *Y_a_* ≠ *Y_b_*) immediately applicable to secure digital signatures.

**Figure 3.**
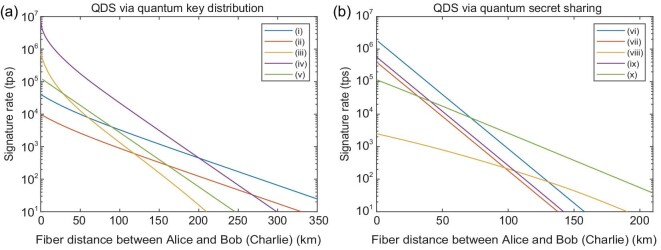
Signature rates versus fiber distances. (a) The rate via quantum key distribution. (b) The rate via quantum secret sharing. Lines labeled (i)–(v) represent quantum key distribution protocols: (i) sending-or-not-sending twin-field protocol [35], (ii) phase-matching protocol [36], (iii) discrete-modulated continuous-variable protocol [37], (iv) Gaussian-modulated continuous-variable protocol [38] and (v) measurement-device-independent protocol [39]. Lines labeled (vi)–(x) represent quantum secret sharing protocols: (vi) prepare-and-measure protocol [40], (vii) measurement-device-independent protocol [41], (viii) round-robin protocol [42], (ix) single-qubit protocol [43] and (x) differential-phase-shift protocol [44]. To simplify, let the channel loss of Alice–Bob and Alice–Charlie be the same. We need a 384-bit key for performing each digital signature with a security bound of approximately 1.1 × 10^−19^ for documents of lengths up to 2^64^. For a gigahertz system, the signature rates are more than 10^4^ tps in the metropolitan area.

Here, we simulate the performances of our OTUH-QDS protocol using various quantum key distribution [35–39] and quantum secret sharing [40–44] protocols (see the online supplementary material), as depicted in Fig. 3. For a gigahertz system and the symmetric channel case, the simulation results show that if the fiber distance between Alice and Bob (or Charlie) is less than 50 km, one can implement digital signatures up to 10^4^ tps, even for the 2^64^-bit document. Therefore, one can conduct tens of thousands of transactions per second secured by digital signatures in a metropolitan area network [12].

Our OTUH-QDS protocol has two significant features. First, as the length of the signed document increases up to 10^19^ bits, the key bits consumed by our protocol are almost constant, while having sufficient security as discussed above. This means that the signature efficiency of our protocol has a significant advantage over previous single-bit-type QDS protocols [20–25,27–31]. As the length of the document is increased from 1 to 10^19^ bits, our quantum resource consumption (384-bit key) does not change, which means that the signature time will not increase. Therefore, our protocol has the signature efficiency advantage from 10^2^ to 10^21^, compared to previous protocols that need at least 10^5^ bits to sign one bit [27–31]. Second, our OTUH-QDS protocol is flexible for all applications. In our QDS protocol, all known and future developed quantum secret sharing and quantum key distribution protocols can be used for the perfect bits correlation of the three parties (secret sharing). Additionally, the universal_2_ hash function should not be restricted to the LFSR-based Toeplitz matrix [45], which is used here just as an example.

### Experimental results of the QDS

**Figure 4.**
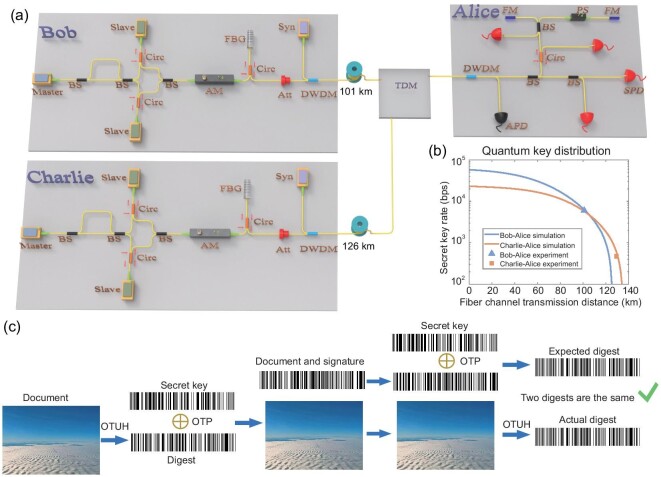
Experimental setup of the quantum secure network. (a) Bob (Charlie) exploits a master laser, an asymmetric interferometer, two slave lasers, two circulators (Circ) and a beam splitter (BS) to prepare optical pulses in the Z and X bases by controlling the trigger electrical signal of slave lasers. The decoy signals are generated by the amplitude modulator (AM), whereas the vacuum state is produced by removing the triggered signal of slave lasers. The optical pulses pass through a set of fiber Bragg grating (FBG) sensors, circulator and attenuator (Att) to be modulated at the single-photon level. The synchronization (Syn) signal is transmitted to quantum channels with a dense wavelength division multiplexer (DWDM). The synchronization pulse is detected by an avalanche photodiode (APD). A biased beam splitter is utilized to realize a passive basis measurement with a single-photon detector (SPD). An asymmetric interferometer is formed by two Faraday mirrors (FM), a phase shifter (PS) and a beam splitter. The quantum signals sent by Bob (Charlie) are received by Alice by time-division multiplexing (TDM). (b) Experimental results of the decoy-state quantum key distribution. The curves of the secret key rate correspond to the simulation results using experimental parameters. (c) Demonstration of quantum digital signatures. The document to be signed with a length of 130 250 bytes includes the timestamp, identity number of the desert image and the image itself.

To verify the efficiency and feasibility of our OTUH-QDS protocol, we established a three-node quantum secure network containing two end nodes (Bob and Charlie) and an intermediate node (Alice), as shown in Fig. 4(a). Two point-to-point quantum key distribution links are built between Alice and Bob and Alice and Charlie using the decoy-state protocol with a time-bin phase encoding system [49]. Bob (Charlie) multiplexes the 1570-nm synchronization pulse with a 1550-nm quantum signal by a dense wavelength division multiplexer, transmitted through a 101-km (126-km) single-mode optical fiber to Alice; the corresponding loss of quantum channels is 19 (24.3) dB, and the system clock frequency is 200 MHz. To reduce the insertion loss of the receiving end, we take advantage of time-division multiplexing by manually switching fiber links. A classic network is used to communicate in the postprocessing stage, including parameter estimation, error correction and privacy amplification (details can be found in the online supplementary material).

In Fig. 4(b), the triangle (square) symbol refers to the experimental secret key rates of quantum key distribution between Bob and Alice (Charlie and Alice) with 6021 (470) bits per second by considering the finite-size effects, fitting well with our simulation curves. The curves are both flattened in the short distance since we introduce dead times of 10 and 25 μs for the gated-mode InGaAs/InP single-photon detector, respectively.

Here, we describe the experimental demonstration of quantum digital signatures for a 130 250-byte (1.042 × 10^6^-bit) document over a 101-km fiber, as shown in Fig. 4(c). The secret sharing is realized so that Alice performs an XOR operation for her two key bit strings. One key bit string is shared with Bob using the time-bin phase encoding quantum key distribution, and the other is shared with Charlie by exploiting another quantum key distribution system. The signed document includes the timestamp, identity number of the desert image and the image itself. The digest is composed of the 128-bit hash value generated through OTUH and the 128-bit irreducible polynomial, and then it is encrypted to form a signature by OTP. Both the digest and signature are displayed as bar codes and have the same size of 32 bytes (256 bits). The actual and expected digests are identical, indicating that we have applied successful quantum digital signatures with information-theoretical security.

For a fair comparison, we also demonstrate the single-bit-type QDS of [23] using the same experimental system. Table 1 shows the results. For signing a single-bit document, the length of the raw key using the method in [23] (without error correction and privacy amplification) is 2.88 × 10^6^ bits. For a multi-bit document, for example, one megabit, at least the length of the key with 2.88 × 10^12^ bits is required [27,32]. Therefore, the signature rate of our OTUH-QDS protocol is 1.22 tps, whereas using the method of Amiri *et al.* [23], it is only 3.23 × 10^−9^ tps if we let the size of each signed document be 10^6^ bits. Fig. 4(c) depicts the experimental demonstration of the QDS. We only require less than one second to run the quantum secure network, whereas using the method of Amiri *et al.* [23], it will take approximately as long as four years to accumulate data.

**Table 1.** List of the experimental results of the QDS in [23] and our OTUH-QDS protocol. At each time, a document of 10^6^ bits is assumed to be signed.

	Amiri *et al.* [23]	Our protocol
Distance between Bob and Charlie (km)	101 + 126 = 227	101 + 126 = 227
Keys consumption (bit)	1.09 × 10^12^ (4.66 × 10^11^)	384
Valid keys length per second (bit)	9314	470
Signature rate (tps)	8.54 × 10^−9^ (2.00 × 10^−8^)	1.22
Security bound (ε)	10^−32^ (10^−10^)	10^−32^

We would like to clarify two main reasons why our protocol shows a huge improvement in the signature efficiency compared with early QDS schemes. First, the early QDS protocols set the threshold value and compare it with the mismatch rate of bit strings that are from the other two parties to determine whether to accept or not. However, after error correction and privacy amplification in our pre-distribution stage, the secret keys of the three parties are perfectly correlated, which satisfies the relationships *X_a_* = *X_b_* ⊕ *X_c_* and *Y_a_* = *Y_b_* ⊕ *Y_c_*. Besides, Bob and Charlie have identical key bit strings instead of partial symmetric key bit strings in previous protocols. This change in key bit strings will result in approximately two orders of magnitude improvement in the signature efficiency due to removing the reception threshold inequality and the corresponding statistical fluctuations. Second, we use the universal_2_ hash function to implement uniform mapping of long documents to short hash values with information-theoretical security. Any attempt to change the document will change the hash value with probability 1 − ε_for_. Since the hash value and universal_2_ hash function are completely unknown (encrypted by a one-time pad), one cannot do anything but randomly guess them. Therefore, we can use a fixed key length to sign documents of almost any length. However, the core of the previous QDS solutions is for the signer to sign the document via a bit-by-bit process [32], which means that one needs at least *m* times the key length to sign an *m*-bit document. Moreover, in previous studies, information-theoretical security has not been proven for signing multi-bit documents. However, in our experiment, it will result in at least a six orders of magnitude improvement in the signature efficiency for the 1.042 × 10^6^-bit-signed document.

### Demonstration of other cryptographic tasks

**Figure 5.**
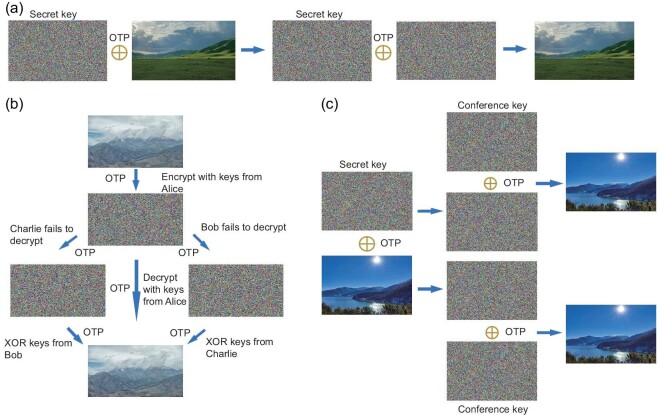
Experimental demonstration of other cryptographic tasks. All encrypted images and secret (conference) keys are demonstrated as images of white noise. (a) Encryption. A prairie image with a size of 112 500 bytes is encrypted via an OTP, utilizing identical secret keys shared between Bob and Charlie, to realize the perfectly private communication. (b) Secret sharing. An image of a mountain with a size of 79 800 bytes is used to realize provable secret sharing. Bob and Charlie can decrypt the image only when they work together to reconstruct the secret keys of Alice. (c) Conference key agreement. An image of a lake with a size of 139 500 bytes is adopted to implement group encryption. All users of this group can obtain the information of the encrypted figure separately.

To demonstrate the full-function information security objectives, shown in Fig. 5, we demonstrate the other three cryptographic tasks in our quantum secure network with information-theoretical security, including encryption, secret sharing and conference key agreement. Fig. 5(a) illustrates quantum private communication with Alice’s help as a trust relay. Alice performs an XOR operation for her two key bit strings that are shared with Bob and Charlie. To realize secure encryption between Bob and Charlie, Alice announces the XOR result as a key relay to make Bob and Charlie share identical keys. To realize quantum communication, a prairie image with 112 500 bytes is encrypted via OTP.

In the secret sharing task, Alice is an honest dealer, while Bob and Charlie are the players. Therefore, either Bob or Charlie is a dishonest player, which can be ensured in quantum key distribution links Charlie–Alice (Bob is the attacker) and Bob–Alice (Charlie is the attacker), respectively. Before the implementation of secure secret sharing, only Alice knows that the XOR result is her key bit string. Quantum secret sharing of an image of a mountain with a size of 79 800 bytes has been implemented, as shown in Fig. 5(b). Only Bob and Charlie cooperate to recover the correct image, while a single player cannot recover the image and only obtains the complete noise map.

For the conference key agreement task, Alice, Bob and Charlie are all honest participants and should have the same keys. This requirement can be realized with information-theoretical security if Alice’s XOR result is published and Charlie changes his key to be the same as Bob’s through an XOR operation. An image of a lake with a size of 139 500 bytes is adopted to implement quantum group encryption, as shown in Fig. 5(c). Any of the three parties can individually obtain the correct image in the group encryption session.

The cryptographic tasks feature high efficiency and information-theoretical security on our quantum secure network using the current quantum technology. We remark that the trusted relay node Alice is required only in the private communication between Bob and Charlie on our quantum secure network. The other three tasks, digital signatures, secret sharing and conference key agreement, are not required since all nodes are the task participants. Note that the secure quantum network may be constructed without a quantum repeater, as proposed in [50].

## CONCLUSIONS

In conclusion, we successfully demonstrated a full-function quantum secure network that meets all information security objectives, namely, confidentiality, integrity, authenticity and non-repudiation. In particular, we theoretically proposed and experimentally implemented an OTUH-QDS protocol that shows a 100-million-fold signature efficiency improvement. As such, digital signatures, which are critical in internet-based digital processing systems, are now promoted to be information-theoretically secure and commercially applicable by OTP, OTUH and secret sharing. Our framework requires few resources to sign an almost arbitrarily long document, outperforming all previous protocols not only in signing efficiency but also in security. Of course, the full-function quantum secure network can be implemented by more advanced technology, such as a future quantum internet. Its successful implementation by a practical quantum secure network under current technology lays a firm foundation for a quantum secure layer of the current internet. Such a quantum secure internet, enabling main secure cryptographic tasks simultaneously, paves the way for the quantum age of the digital economy.

## METHODS

### Pre-distribution stage

The pre-distribution stage ensures that each participant has two key bit strings and meets the secret sharing relationships *X_a_* = *X_b_* ⊕ *X_c_* and *Y_a_* = *Y_b_* ⊕ *Y_c_*, which can be realized using quantum communication protocols with information-theoretical security.

There are two quantum key distribution links if quantum key distribution protocols are being used. The Bob–Alice (Charlie–Alice) link will generate the symmetric quantum keys, denoted as $S_{ba}^{b}=S_{ba}^{a}$$(S_{ca}^{c}=S_{ca}^{a})$, and even dishonest Charlie (Bob) has no knowledge about it. Alice implements an XOR operation to obtain her new quantum key $S^{a}=S_{ba}^{a}\oplus S_{ca}^{a}$. Therefore, since Alice has all the knowledge of Bob’s and Charlie’s keys, she can only be the signer, while Bob and Charlie can be the receivers. We remark that the XOR operation of Alice generates asymmetry between Alice and Bob.

Alice, Bob and Charlie can directly generate the perfect correlation quantum keys *S_a_* = *S_b_* ⊕ *S_c_* if quantum secret sharing protocols are being used. Traditional quantum secret sharing protocols require that the dealer Alice is honest and that the player Bob or Charlie can be allowed to be dishonest. The dishonest Bob and Charlie do not know *S_a_* and they can be the receiver. Note that since Alice can obtain all information of *S_b_* and *S_c_* if she is dishonest in performing traditional quantum secret sharing, Alice cannot be a receiver if traditional quantum secret sharing protocols are being used. However, if one adopts measurement-device-independent quantum secret sharing [41], all three participants will not know any information about the others’ quantum keys; anyone of them can be a receiver or signer.

### One-time universal_2_ hash function

A collection *H* of hash functions *h*: $\mathbb {S}$→$\mathbb {T}$ is said to be universal_2_ [34] if, for every two different $x, y\in \mathbb {S}$, we have


(2) \begin{equation*} {\rm Pr}_{h\in H}[h(x)=h(y)]\le \frac{1}{|\mathbb {T}|}. \end{equation*}

This means that the universal_2_ hash function can uniformly map the long documents to short hash values with a small collision probability. The random matrices belong to the universal_2_ hash functions, which require *mn* random bits for specifying hash functions (seen as an *mn* Boolean matrix) to transform the *m*-bit document into an *n*-bit hash value. To reduce the cost of random bits, the Toeplitz matrix [2], which requires only *m* + *n* − 1 random bits, is widely used in randomness extraction and privacy amplification, and its collision probability is 1/2^*n*^. Nevertheless, it still requires the length of random input bits to be longer than that of the document. Fortunately, the LFSR-based Toeplitz matrix [45] is the almost universal_2_ hash function, where the hash function is determined by an irreducible polynomial *p*(*x*) of degree *n* over the Galois field GF(2) and *n*-bit random initial vector. The collision probability of the LFSR-based Toeplitz matrix [45] is *m*/2^*n* − 1^ (see the online supplementary material). The initial vector and irreducible polynomial of the LFSR-based Toeplitz matrix are randomly changed for each signature, which is an important and novel requirement of our OTUH-QDS protocol.

## DATA AVAILABILITY

The data that support the findings of this study are available from the corresponding author upon reasonable request.

## Supplementary Material

nwac228_Supplemental_File
