# Supplementary material for: Experimental quantum secure network with digital signatures and encryption
Source: Natl Sci Rev. 2022 Oct 22;10(4):nwac228. doi: 10.1093/nsr/nwac228 (PMC10165682; doi:10.1093/nsr/nwac228)
Supplement: nwac228_Supplemental_File [file nwac228_supplemental_file.pdf]

Supplementary data for
Experimental quantum secure network with digital signatures and encryption

Hua-Lei Yin,[1,*] Yao Fu,[2,†] Chen-Long Li,[1] Chen-Xun Weng,[1] Bing-Hong Li,[1] Jie Gu,[1] Yu-Shuo Lu,[1] Shan Huang,[1] and Zeng-Bing Chen[1,2,‡]

[1]*National Laboratory of Solid State Microstructures and School of Physics, Collaborative Innovation Center of Advanced Microstructures, Nanjing University, Nanjing 210093, China*
[2]*MatricTime Digital Technology Co. Ltd., Nanjing 211899, China*
(Dated: September 15, 2022)

1. Cryptography toolbox

Here, we introduce threats faced by information processing (as shown in Fig. S1), the corresponding information security objectives and classical cryptographic techniques to tackle such threats [1]. First, eavesdropping, which threatens the confidentiality of information, can be prevented using symmetric cryptography and asymmetric cryptography (i.e., public-key cryptography). Second, tampering, which destroys the integrity of data, can be approached with a one-way hash function, message authentication code and digital signatures. Third, disguise, in which the attacker pretends to be the real information sender, can deal with message authentication codes and digital signatures. Finally, one may repudiate his or her certain behavior, and the digital signatures provide the efficacy of non-repudiation.

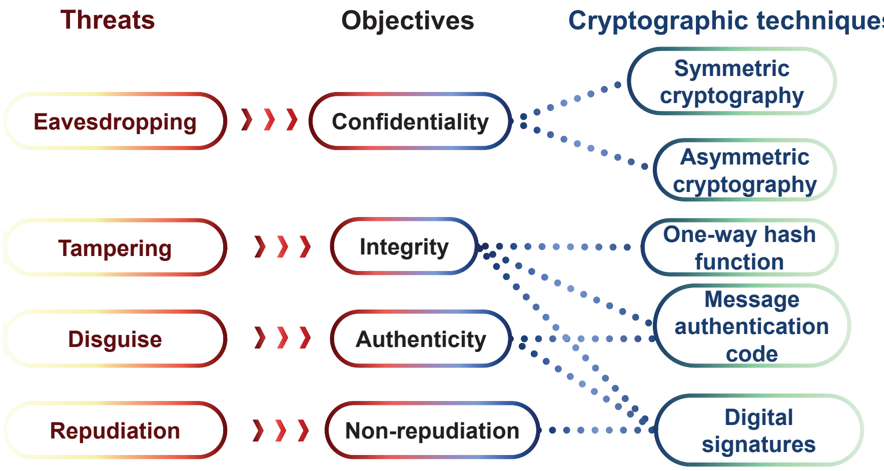

Supplementary Figure S1. **Cryptography toolbox.**

2. One-time pad

Define that encryption algorithm E maps plaintexts $m \in M$ to ciphertexts $c \in C$. According to Shannon's information theory, an algorithm E is perfectly secret if C and M are independent, i.e., $\Pr(m, c) = \Pr(m) \times \Pr(c)$. The one-time pad [2] encryption satisfies the above definition, where a plaintext is paired using the XOR operation with a random secret key of the same length. Learning the ciphertext does not increase any plaintext information. In addition, each encryption requires a new and independent random key; hence, knowing details about the previous key does not help the attacker.

In our one-time universal$_2$ hashing quantum digital signatures (OTUH-QDS) scheme, we utilize the one-time pad to encrypt the hash value and the irreducible polynomial to acquire the signature so that we can completely conceal the information of (almost) universal$_2$ hash functions.

3. LFSR-based Toeplitz hashing and message authentication

The LFSR-based Toeplitz matrix [3] is the almost universal$_2$ hash function, where the hash function is determined by an irreducible polynomial $p(x) = x_n + a_{n-1}x^{n-1} + ... + a_1x + a_0$ of degree n over the Galois field GF(2) and n-bit random initial vector, just requiring a total of $2n$ bits. The randomness of the initial vector and irreducible polynomial

together guarantees the security of LFSR-based Toeplitz hashing. The collision probability of LFSR-based Toeplitz hashing is bounded by $\epsilon = m/2^{n-1}$, which quantifies the upper bound on the probability of finding any two different documents with the same hash value. The LFSR-based Toeplitz hashing operation can be written as $h_{p,s}(M)$= $H_{nm} \cdot M = Hash$, where $p = (a_{n-1}, a_{n-2}, ..., a_1, a_0)$ represents an irreducible polynomial and $s = (b_n, b_{n-1}, ..., b_2, b_1)^T$ is the initial vector. p and s are random and determine the Toeplitz matrix H_{nm} with n rows and m columns. $M = (M_1, M_2...M_m)^T$ is the message in the form of an m-bit vector. The Toeplitz matrix can be written as $H_{nm} = (s, s_1, ..., s_{m-1})$, where the initial vector $s = (b_n, b_{n-1}, ..., b_2, b_1)^T$ is the first column. The LFSR, determined by p, will be performed $m-1$ times to generate s_1, s_2, ..., s_{m-2} and s_{m-1}. To be specific, LFSR will shift down every element in the previous column, and add a new element to the top of the column. For example, LFSR will first transform s into $s_1 = (b_{n+1}, b_n, ..., b_3, b_2)^T$, where $b_{n+1} = p \cdot s$, and likewise, transform s_1 to s_2. Then the m vectors $s, s_1, ..., s_{m-1}$ will together construct the Toeplitz matrix $H_{nm} = (s, s_1, ..., s_{m-1})$, and the hash value of the message is $H_{nm} \cdot M = Hash$.

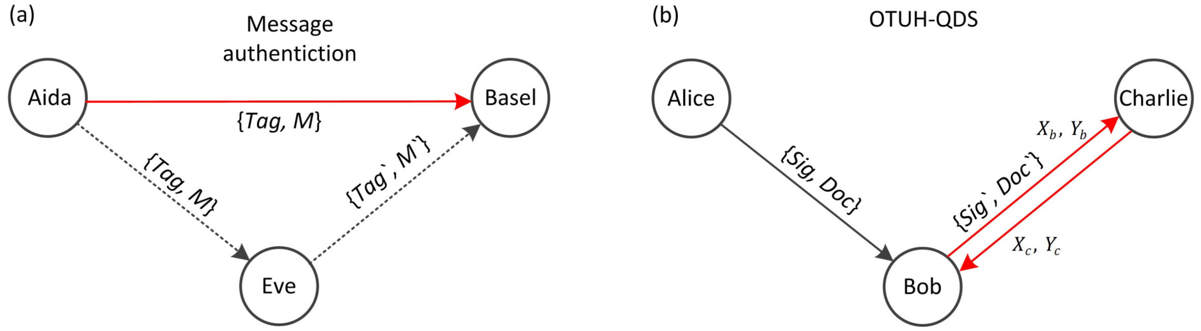

Supplementary Figure S2. **Comparing message authentication and digital signatures.** (a) Information-theoretically secure message authentication. Aida and Basel are the message sender and recipient, respectively, while Eve is the attacker. Eve tries to make Basel accept the tampered tag and message $\{Tag', M'\}$. The black dotted line represents Eve's attack process. (b) OTUH-QDS. For Bob's forgery attack, he tries to make Charlie accept the forged siganture and document $\{Sig', Doc'\}$. The red solid line represents the authentication classical channel, which has information-theoretical security by performing message authentication.

Failure probability of authentication.—Here, we first provide the information-theoretical security proof of message authentication [3]. We remark that the security proof in the original literature is omitted [3], we provide a detailed proof here, and the conclusions are consistent. Suppose the message authentication scenario that an attacker Eve captures a tag and message $\{Tag,\ M\}$ from the sender Aida, as shown in Fig. S2a. Note that the tag is acquired by using random secret key Key to encrypt hash value $Hash$ with a one-time pad, i.e., $Tag = h_{p,s}(M) \oplus Key$. Eve cannot obtain any information of the Toeplitz hash function and the hash value due to the one-time pad. Eve can only randomly guess the initial vector s and irreducible polynomial p. Eve can tamper with the message successfully if and only if he guesses a combination $\{t,\ m\}$ with a very small probability that meets the relationship $h_{p,s}(m) = t$. Eve sends a new (tampered) tag and message $\{Tag' = Tag \oplus t,\ M' = M \oplus m\}$ to the recipient Basel. In this case, the recipient will accept the message because of the relationship $h_{p,s}(M \oplus m) = h_{p,s}(M) \oplus h_{p,s}(m) = Tag \oplus t \oplus Key$. It must be mentioned that $m \neq 0$ due to the requirement of a valid forge. Thus, the failure probability $\epsilon_{\rm aut}$ of authentication by using LFSR-based Toeplitz hashing can be defined by the probability of one successfully choosing a combination $\{t,\ m\}$ with the relationship $h(m) = t$ and $m \neq 0$,

$$\epsilon_{\rm aut} = \Pr[h_{p,s}(m) = t,\ m \neq 0]. \tag{1}$$

We remark that the attacker Eve does not have any prior information about p and s and can only guess randomly. Moreover, before Eve sends the tag and message to the recipient, Eve also cannot be sure if he guessed correctly even if he exhausts all the possibilities, which is with the information-theoretical security [3]. To quantify the failure probability $\epsilon_{\rm aut}$, we need to consider two cases, $t = 0$ and $t \neq 0$. Note that $h_{p,s}(M)$ can be rewritten as

$$h_{p,s}(M) = h_{p,M}(s) = \sum_{i=0}^{m-1} M_{i+1} W^i \cdot s, \tag{2}$$

where W is the $n \times n$ circulant matrix with the first row being p. Denote the characteristic value of W as λ_i $(i = 1, 2, 3, ..., n)$. One can diagonalize the matrix $\sum_{i=0}^{m-1} M_{i+1} W^i$, and the diagonal elements are $m(\lambda_i)$. After

calculation, it can be verified that $p(x)$ is just the characteristic polynomial of W, which means that there is the relationship $p(\lambda_i) = 0$. For the first case $h_{p,M}(s) = t = 0$, since $s \neq 0$, $h_{p,M}(s) = 0$ only if there exists a zero diagonal element in the diagonalized matrix, i.e., it has a zero characteristic value. Thus, there exists a λ_i satisfying $m(\lambda_i) = 0$. This is equivalent to $p(x)|m(x)$ since $p(\lambda_i) = 0$. The probability of such an event is at most the number of possible irreducible factors of $M(x)$ divided by the total number of irreducible polynomials of degree n. The former is at most m/n, and the latter is at least $2^{n-1}/n$ [4]. Thus, the probability of $h_{p,s}(m) = t = 0$ is at most $(m/n)/(2^{n-1}/n) = m/2^{n-1}$. We include all the cases in which there exists a λ_i satisfying $m(\lambda_i) = 0$ in the first case, which is equivalent to the fact that the rank of $\sum_{i=0}^{m-1} M_{i+1}W^i$ is less than n. Thus, for the second case $h_{p,M}(s) = t \neq 0$, the rank of $\sum_{i=0}^{m-1} M_{i+1}W^i$ is equal to n. This means that $h_{p,M}(s) = \sum_{i=0}^{m-1} M_{i+1}W^i \cdot s$ is a bijection, i.e., one-to-one mapping. There are $2^n - 1$ possible values of $s \neq 0$ corresponding to the $2^n - 1$ different tag values, so the probability of $h_{p,s}(m) = t \neq 0$ is $1/(2^n - 1)$. Therefore, the upper bound on the failure probability of message authentication using LFSR-based Toeplitz hashing is [3]

$$\epsilon_{\text{aut}} = \max\left\{\frac{1}{2^n - 1}, \frac{m}{2^{n-1}}\right\} = \frac{m}{2^{n-1}}. \tag{3}$$

We remark that message authentication is the premise and foundation for realizing the information-theoretical security of quantum key distribution [5], where the basis sift, error verification, and privacy amplification steps all require message authentication to ensure the information-theoretical security.

4. Secure OTUH-QDS against Bob's forgery attack

Now, we will show that Bob's forgery attack in our OTUH-QDS protocol will be related to Eve's attack in secure message authentication, as shown in Fig. S2b. These two types of attacks are different but have many correlations. In message authentication, the message sender Adia and recipient Basel are honest and trust each other all the time. They will get together to against Eve's attack. A successful attack in message authentication is when Eve changes the tag and message and the recipient will accept it. In our OTUH-QDS, there are two types of Bob's forgery attacks since the signer Alice cannot be regarded as always honest. The first type is that Bob can generate a new signature and document if Alice has not signed a document at all. The second type is that Bob can change the signature and document if Alice has signed a document. Note that Bob does not have any information about the initial vector and the irreducible polynomial before he forwards the signed signature and document to charlie, which is the same as Eve in message authentication. We assume that the information-theoretically secure message authentication and OTUH-QDS will be performed many rounds to transfer multiple messages and sign multiple documents, respectively. Obviously, for the first round, the initial vector and irreducible polynomial are new and random. The failure probability is the same between Eve's attack in message authentication and Bob's forgery attack with the second type in OTUH-QDS since the attacker's purpose and the conditions for success are exactly the same, i.e., the failure probability are both $m/2^{n-1}$.

However, there is an important difference between message authentication and OTUH-QDS in the second round and beyond. In message authentication (Adia and Basel are always honest), the input initial vector and the irreducible polynomial can be fixed [5, 6] in later rounds since the attacker Eve cannot know the initial vector and the irreducible polynomial after message authentication has been performed. Bob also does not have any information about the initial vector and the irreducible polynomial before forwarding the signed document and signature to charlie. However, Charlie will forward his key bit strings to Bob after Charlie receives the document and signature forwarded by Bob. Thus, Bob can obtain all the information of the initial vector and the irreducible polynomial after the implementation of each round of digital signatures. To forbid Bob to exploit the information from the previous round to implement the attack. An important observation in our OTUH-QDS protocol is that the initial vector and the irreducible polynomial cannot be fixed and must be randomly updated in every round. In other words, the universal$_2$ hash function will only be used once and then be updated. Thus, we denote it as one-time universal$_2$ hashing, which is similar to the one-time pad.

5. Algorithms for testing irreducibility of polynomials over GF(2)

Irreducible polynomial.—A crucial point is that in every round, the signer must randomly choose an irreducible polynomial in our OTUH-QDS scheme. In our protocol, Alice finishes this task with an n-bit quantum random number. Denote the format of the irreducible polynomial as $p(x) = x^n + a_{n-1}x^{n-1} + \cdots + a_1x + a_0$, where $a_i = 0$ or 1. Then $p(x)$ can be characterized by an n-bit string $p = (a_{n-1}, a_{n-2}, \cdots, a_1, a_0)$, i.e., every bit of the string determines a corresponding coefficient of $p(x)$. To generate the irreducible polynomial, Alice first uses the n-bit random number to generate a polynomial $p_1(x)$, and checks out whether $p_1(x)$ is irreducible. If $p_1(x)$ is irreducible,

it will be used to generate the LFSR-based Toeplitz matrix. Otherwise, Alice utilizes a new n-bit random number to obtain a new string and generates a new polynomial and then examines whether it is an irreducible polynomial. Alice will repeat this step until she generates an irreducible polynomial. For example, choose $n = 128$. Assume Alice first generates $p_1(x) = x^{128} + x^{29} + x^{25} + x + 1$. Then, she will find it reducible and successively generate and examine $p_2(x) = x^{128} + x^{50} + x^{27} + x^2 + 1$ and $p_3(x) = x^{128} + x^{29} + x^{27} + x^2 + 1$. Finally, she finds $p_3(x)$ irreducible and chooses $p_3(x)$ as the irreducible polynomial to generate the Toeplitz matrix. Note that for an irreducible polynomial $p(x)$, one always has $a_0 = 1$. Thus, we can use $n-1$ random bits $(a_{n-1}, a_{n-2}, \cdots, a_1, 1)$ to generate the polynomial. We remark that the quantum random number used for generating the irreducible polynomial is produced locally by Alice's quantum random number generator.

The test algorithm.—The following is the concrete method we employed to check whether a polynomial over GF(2) is irreducible or not. Suppose $p(x)$ is a polynomial of order n in GF(2). According to [7], the necessary and sufficient condition for $p(x)$ being irreducible is:

$$\begin{cases} (A)\ x^{2^n} \equiv x \mod p(x) \\ (B)\ \gcd\left(x^{2^{\frac{n}{d}}} - x,\ p(x)\right) = 1, \end{cases} \tag{4}$$

where d is any prime factor of n and $\gcd(f(x),\ g(x))$ represents the greatest common divisor (GCD) of $f(x)$ and $g(x)$. In our scheme, $n = 128 = 2^7$, i.e., n only has one prime factor "2". Thus, to verify condition (2), we just need to examine $\gcd\left(x^{2^{64}} - x, p(x)\right) = 1$. To speed up our calculation, we utilize fast modular composition (FMC) algorithms and an extended Euclidean algorithm [8]. The FMC algorithms can calculate $x^{2^{128}}$ and $x^{2^{64}}$ mod $p(x)$ with high speed by calculating $x^{2^{2^7}}$ and $x^{2^{2^6}}$, while the extended Euclidean algorithm can quickly finish the GCD calculation. Thus, conditions (A) and (B) can be verified efficiently. In our simulation, we search 1000 irreducible polynomials, and on average, it takes 73.6 tests to find an irreducible polynomial, consistent with the conclusion [3] that the total number of irreducible polynomials of order n in GF(2) is at least $2^{n-1}/n$ and at most $2^n/n$ [4]. We implement our simulation in a desktop with an Intel i5-10400 CPU (with RAM of 8 GB), and on average, it takes approximately 0.36 seconds to generate an irreducible polynomial of order 128 from a 128-bit random input.

6. Numerical simulation of our QDS via quantum key distribution element

Considering the case that we have two quantum key distribution links between Alice-Bob and Alice-Charlie, the two links have the same loss. Alice and Bob share one set of secret keys S_1, while Alice and Charlie share the other set of secret keys S_2 by implementing quantum key distribution (QKD) with the key rate R_{QKD}. For the requirement of secret sharing, Alice, Bob and Charlie utilize K_a, K_b and K_c as their correlation keys, respectively, where $K_a = S_1 \oplus S_2$, $K_b = S_1$ and $K_c = S_2$. Therefore, the signature rate can be defined as $R_{\mathrm{QDS}} = R_{\mathrm{QKD}}/3n$, since one needs a $3n$-bit key to implement OTUH and OTP in our OTUH-QDS protocol.

(6.1) Sending-or-not-sending twin-field quantum key distribution [9].
The secure key rate is given by

$$R_{\mathrm{QKD}} = 2t(1-t)\mu e^{-\mu} Y_1 \left[1 - H\left(e_1^{\mathrm{ph}}\right)\right] - Q_Z f H(E^Z), \tag{5}$$

where t is the probability of sending the signal pulse at a signal window, μ is the intensity of the signal pulse, Y_1 is the counting rate of Z_1-windows, Q_Z is the observed counting rate of Z-windows, $e_1^{\mathrm{ph}} = e_1^{X_1}$ is the phase-flip error rate for Z_1-windows, E^Z is the bit error rate for Z-windows, and f is the error correction efficiency. The yield Y_1 and error rate $e_1^{X_1}$ can be estimated by exploiting the decoy-state method,

$$Y_1 \geq \frac{\mu/2}{\mu\nu - \nu^2} \left[e^{\nu}(Q_{\nu 0} + Q_{0\nu}) - \frac{\nu^2}{\mu^2} e^{\mu}(Q_{\mu 0} + Q_{0\mu}) - 2\frac{\mu^2 - \nu^2}{\mu^2} Q_{00} \right], \tag{6}$$

$$e_1^{X_1} \leq \frac{1}{2\nu Y_1}\left(e^{2\nu} E_{\nu\nu}^{\mathrm{pm}} Q_{\nu\nu}^{\mathrm{pm}} - \frac{1}{2} Y_0 \right), \tag{7}$$

where ν is the intensity of the decoy pulse, Q_{k_a,k_b} is the gain when Alice chooses intensity k_a and Bob chooses intensity k_b ($k_a,\ k_b \in \{\mu,\ \nu,\ 0\}$), $E_{\nu\nu}^{\mathrm{pm}}$ is the bit error rate, $Q_{\nu\nu}^{\mathrm{pm}}$ is the gain of intensity ν for both Alice and Bob after successful postselected phase-matching and $Y_0 = Q_{00}$ is the counting rate of the vacuum state.

The gain Q_{k_a,k_b} can be given as

$$Q_{k_a,k_b} = 2(1-p_d)e^{-\frac{k_a+k_b}{2}\sqrt{\eta}}\left[I_0\left(\sqrt{k_a k_b}\sqrt{\eta}\right) - (1-p_d)\,e^{-\frac{k_a+k_b}{2}\sqrt{\eta}}\right], \tag{8}$$

where $\eta = \eta_d^2 \times 10^{-\alpha L/10}$ is the total efficiency and η_d, α and L are the detector efficiency, attenuation coefficient and distance, respectively. p_d is the dark count rate, and $I_0(x)$ is the modified Bessel function of the first kind. The gain $Q_{\nu\nu}^{\mathrm{pm}}$ and bit error rate $E_{\nu\nu}^{\mathrm{pm}}$ can be given by

$$Q_{\nu\nu}^{\mathrm{pm}} = Q_{c,\nu\nu}^{\mathrm{pm}} + Q_{e,\nu\nu}^{\mathrm{pm}}, \tag{9}$$

$$E_{\nu\nu}^{\mathrm{pm}} = \left[e_d^x \mathrm{Q}_{c,\nu\nu}^{pm} + (1-e_d^x)\,Q_{e,\nu\nu}^{pm}\right]/\mathrm{Q}_{\nu\nu}^{pm}, \tag{10}$$

where e_d^x is the misalignment rate of the X basis. Therein, the correct gain $Q_{c,\nu\nu}^{\mathrm{pm}}$ and incorrect gain $Q_{e,\nu\nu}^{\mathrm{pm}}$ are

$$Q_{c,\nu\nu}^{\mathrm{pm}} \approx \frac{1-p_d}{\delta}\sqrt{\frac{\pi}{2\nu\sqrt{\eta}}}\,\mathrm{erf}\left(\sqrt{\frac{\nu\sqrt{\eta}}{2}}\delta\right) - (1-p_d)^2\,e^{-2\nu\sqrt{\eta}}, \tag{11}$$

$$Q_{e,\nu\nu}^{\mathrm{pm}} \approx \frac{1-p_d}{\delta}e^{-2\nu\sqrt{\eta}}\sqrt{\frac{\pi}{2\nu\sqrt{\eta}}}\,\mathrm{erfi}\left(\sqrt{\frac{\nu\sqrt{\eta}}{2}}\delta\right) - (1-p_d)^2\,e^{-2\nu\sqrt{\eta}}, \tag{12}$$

where $\mathrm{erf}(x)$ and $\mathrm{erfi}(x)$ are the error function and imaginary error function, respectively. The gain Q_Z and bit error rate E^Z can be given by

$$Q_Z = (1-t)^2 Q_{00} + 2t(1-t)Q_{0\mu} + t^2 Q_{\mu\mu}, \tag{13}$$

and

$$E^Z = \frac{(1-t)^2 Q_{00} + t^2 Q_{\mu\mu}}{(1-t)^2 Q_{00} + t(1-t)(Q_{0\mu} + Q_{\mu 0}) + t^2 Q_{\mu\mu}}. \tag{14}$$

In the simulation, we set $p_d = 10^{-8}$, $f = 1.1$, $\eta_d = 85\%$, $e_d = 2\%$ and $\alpha = 0.167$ dB/km. The light intensities and probability t are globally optimized.

(6.2) Phase-matching quantum key distribution [10].
Here we present the simulation details of the phase-matching quantum key distribution. The overall secure key rate is given by

$$R_{\mathrm{QKD}} = \frac{2}{D}Q_\mu[1 - h(E^{\mathrm{ph}}) - fh(E^{\mathrm{b}})], \tag{15}$$

where Q_μ is the total gain of the signal pulses, $2/D$ is the phase-sifting factor with $D = 16$, f is the error correction efficiency, $h(x) = -x\log_2 x - (1-x)\log_2(1-x)$ is the binary entropy function, E^{ph} is the phase error rate and E^{b} is the bit error rate. The overall phase-error rate [11] is bounded by $E^{\mathrm{ph}} \le 1 - q_1$, where $q_1 = \mu e^{-\mu} Y_1/Q_\mu$. By using the decoy state method with three intensities, the yield of the single-photon component can be given as

$$Y_1 \ge \frac{\mu}{\mu\nu - \nu^2}\left(Q_\nu e^\nu - Q_\mu e^\mu \frac{\nu^2}{\mu^2} - \frac{\mu^2 - \nu^2}{\mu^2}Q_0\right). \tag{16}$$

For simulation, the gain of intensity k ($k \in \{\mu,\ \nu,\ 0\}$) is $Q_k = 1 - (1-2p_d)e^{-k\eta}$. The bit error rate is $E^{\mathrm{b}} = [p_d + \eta\mu(e_\Delta + e_d)]\frac{e^{-\eta\mu}}{Q_\mu}$, where p_d is the dark count rate, $\eta = \eta_d \times 10^{-\alpha L/20}$ is the channel transmittance, e_d is the extra misalignment error and the intrinsic error $e_\Delta = \sin^2(\frac{\pi}{D})$. In the simulation, we set $p_d = 10^{-8}$, $f = 1.1$, $\eta_d = 85\%$, $\alpha = 0.167$ dB/km and $e_d = 2\%$. The intensities μ, ν are globally optimized.

(6.3) Discrete-modulated continuous-variable quantum key distribution [12].
In the case of reverse reconciliation, the secret key rate under collective attacks in the asymptotic limit is given by $R_{\mathrm{QKD}} = p_{pass}[I(X;Z) - \max_{\rho\in\mathbf{S}}\chi(Z:E)]$, where $I(X;Z)$ is the classical mutual information between Alice's string

X and the raw key string Z, $\chi(Z:E)$ is the information of Eve's knowledge about the raw key string Z and $p_{pass} = 1-\frac{1}{2}\int_{-\Delta_c}^{\Delta_c} P(q|0)dq - \frac{1}{2}\int_{-\Delta_c}^{\Delta_c} P(q|1)dq$ is the sifting probability. $P(q|0)$ and $P(q|1)$ are probability distributions of Bob's homodyne detection conditioned that Alice generates bit 0 or 1. The set $\mathbf{S}$ contains all density operators compatible with experimental observations. According to [12], this key rate can be reformulated as a convex optimization problem, and the secret key rate is $R = \min_{\rho\in\mathbf{S}} D(\mathcal{G}(\rho)||\mathcal{Z}[\mathcal{G}(\rho)]) - p_{pass}\delta_{EC}$, where $D(\rho||\sigma) = \mathrm{Tr}(\rho\log_2\rho) - \mathrm{Tr}(\rho\log_2\sigma)$ is the quantum relative entropy. $\mathcal{G}$ is a map describing classical postprocessing. For the reverse reconciliation, we have $\mathcal{G}(\sigma) = K\sigma K^\dagger$, where $K = \sum_{z=0}^{1} |z\rangle_R \otimes (|0\rangle\langle 0| + |1\rangle\langle 1|)_A \otimes (\sqrt{I_z})_B$ with $I_0 = \int_{\Delta_c}^{\infty} dq|q\rangle\langle q|$ and $I_1 = \int_{-\infty}^{-\Delta_c} dq|q\rangle\langle q|$. We set $\Delta_c = 0$ in our simulation. $\mathcal{Z}$ is a pinching quantum channel, and we have $\mathcal{Z}(\rho) = \sum_j Z_j\rho Z_j$, where $Z_0 = |0\rangle\langle 0|_R \otimes \mathcal{I}_{AB}$ and $Z_1 = |1\rangle\langle 1|_R \otimes \mathcal{I}_{AB}$. $\delta_{EC} = (1-\beta)H(Z) + \beta h(e)$ is the amount of information leakage per signal, where $e = \frac{1}{p_{pass}}\left(\frac{1}{2}\int_{\infty}^{-\Delta_c} P(q|0)dq + \frac{1}{2}\int_{\Delta_c}^{\infty} P(q|1)dq\right)$. The convex optimization problem can be described as follows:

$$\begin{aligned}
&minimize \quad D(\mathcal{G}(\rho)||\mathcal{Z}[\mathcal{G}(\rho)])\\
&subject \quad to\\
&\qquad Tr[\rho(|i\rangle\langle i|_A \otimes \hat{q})] = p_x\langle\hat{q}\rangle_i,\\
&\qquad Tr[\rho(|i\rangle\langle i|_A \otimes \hat{p})] = p_x\langle\hat{p}\rangle_i,\\
&\qquad Tr[\rho(|i\rangle\langle i|_A \otimes \hat{n})] = p_x\langle\hat{n}\rangle_i,\\
&\qquad Tr[\rho(|i\rangle\langle i|_A \otimes \hat{d})] = p_x\langle\hat{d}\rangle_i,\\
&\qquad Tr[\rho] = 1,\\
&\qquad \rho \geqslant 0,\\
&\qquad Tr_B[\rho] = \sum_{i,j=0}^{3} \sqrt{p_i p_j}\langle\phi_j|\phi_i\rangle|i\rangle\langle j|_A,
\end{aligned} \tag{17}$$

where $\hat{n} = \frac{1}{2}(\hat{q}^2 + \hat{p}^2 - 1) = \hat{a}^\dagger\hat{a}$ and $\hat{d} = \hat{q}^2 - \hat{p}^2 = \hat{a}^2 + (\hat{a}^\dagger)^2$. We consider a quantum channel with transmittance $\eta = 10^{-0.167L/10}$ where L is the distance and excess noise $\xi = 0.01$. In such a model, we have $P(q|0) = \frac{1}{\sqrt{\pi(1+\eta\xi)}}e^{-\frac{[\alpha-\sqrt{2\eta}q]^2}{1+\eta\xi}}$ and $P(q|1) = \frac{1}{\sqrt{\pi(1+\eta\xi)}}e^{-\frac{[\alpha+\sqrt{2\eta}q]^2}{1+\eta\xi}}$. The expectation values can be given by

$$\begin{aligned}
\langle\hat{q}\rangle &= \sqrt{2\eta}Re(\alpha),\\
\langle\hat{p}\rangle &= \sqrt{2\eta}Im(\alpha),\\
\langle\hat{n}\rangle &= \eta|\alpha|^2 + \frac{\eta\xi}{2},\\
\langle\hat{d}\rangle &= \eta[\alpha^2 + (\alpha^*)^2].
\end{aligned} \tag{18}$$

α is the amplitude of the signal, and we optimize α in the interval of $[0.35, 0.6]$ with a step of 0.01.

(6.4) Gaussian-modulated continuous-variable quantum key distribution [13].
From an information-theoretic point of view, in the case of reverse reconciliation, Alice and Bob can distill perfectly correlated secret key bits provided that the amount of information they share I_{AB} is higher than the information acquired by Eve χ_{BE} under collective attacks. Therefore, the secret key rate is defined as $R = I_{AB} - \chi_{AB}$. Considering the inefficiency of error correction, a factor β is introduced as the reconciliation efficiency, and the secret key rate formula is $R_{\mathrm{QKD}} = \beta I_{AB} - \chi_{AB}$. In our numerical simulation, we use $\beta = 0.95$. We calculate the mutual information using

$$I_{AB} = \frac{1}{2}\log_2\frac{V + \chi_{\mathrm{tot}}}{1 + \chi_{\mathrm{tot}}}, \tag{19}$$

where V is the variance of the thermal state observed at Alice's lab, and χ_{tot} is the total excess noise added between Alice and Bob. Here, we detail the formula of χ_{tot} as follows:

$$\chi_{tot} = \frac{1}{\eta T(1 + v_{el}) + \xi - 1}, \tag{20}$$

where T is the transmission of the quantum channel, ξ is the excess noise, v_{el} is the electronic noise and η is the detection losses. To draw the line, we set $\eta = 0.85$, $v_{\mathrm{el}} = 0$, $\xi = 0.01$, $T = 10^{-0.167L/10}$ and V is optimized. L is

the distance. Under collective attacks, Eve's accessible information is upper bounded by the Holevo quantity χ_{BE} satisfying

$$\chi_{BE}=G\left(\frac{\lambda_1-1}{2}\right)+G\left(\frac{\lambda_2-1}{2}\right)-G\left(\frac{\lambda_3-1}{2}\right)-G\left(\frac{\lambda_4-1}{2}\right), \tag{21}$$

where $G(x)=(x+1)\log_2(x+1)-x\log_2 x$, and $\{\lambda_i\}$ are the symplectic eigenvalues of the covariance matrix characterizing the quantum state. Calculation shows that $\lambda_{1,2}^2=\frac{1}{2}[A\pm\sqrt{A^2-4B}]$, where $A=V^2(1-2T)+2T+T^2(V+\xi-1+\frac{1}{T})^2$ and $B=T^2(V/T-V+V\xi+1)^2$ and $\lambda_{1,2}^2=\frac{1}{2}[C\pm\sqrt{C^2-4D}]$, where $C=\frac{V\sqrt{B}+T(V+1/T-1+\xi)+A/\eta(1+v_{\rm el})-A}{T(V+\chi_{\rm tot})}$ and $D=\sqrt{B}\frac{V+\sqrt{B}[1/\eta(1+v_{\rm el})-1]}{T(V+\chi_{\rm tot})}$.

(6.5) Measurement-device-independent quantum key distribution [14].
For the measurement-device-independent quantum key distribution, we consider Alice and Bob sending a coherent state and the ideal case where only the channel loss and detector efficiency are taken into consideration. The secret key rate is given by

$$R_{\rm QKD}=\frac{1}{2e^2}\eta^2, \tag{22}$$

where e is the natural constant, $\eta=\eta_d\times 10^{-\alpha L/20}$ is the total channel transmittance and L is the distance between Alice and Bob. We set $\eta_d=85\%$, $\alpha=0.167$ dB/km.

7. Numerical simulation of our QDS via quantum secret sharing element

Different from quantum key distribution, quantum secret sharing can directly offer secret sharing correlation with unconditional security via quantum laws. In quantum secret sharing of three users, Alice is the dealer, while both Bob and Charlie are players. Even though one of Bob and Charlie is dishonest, they can also generate the secure correlation key $K_a=K_b\oplus K_c$. We assume that the distances of Alice-Bob and Alice-Charlie are the same. The signature rate can be defined as $R_{\rm QDS}=R_{\rm QSS}/3n$, since one needs a $3n$-bit key for OTUH and OTP.

7.1) Prepare-and-measure quantum secret sharing [15].
Using the Greenberger-Horne-Zeilinger (GHZ) state can realize quantum secret sharing directly. Here, we consider an equivalent prepare-and-measure protocol. In our simulation, we only consider the photon loss in the quantum channel and detector. Therefore, the secret key rate is given by

$$R_{\rm QSS}=\eta^2, \tag{23}$$

where $\eta=\eta_d\times 10^{-\alpha L/10}$, $\eta_d=85\%$ and $\alpha=0.167$ dB/km. L is the distance between Alice and Bob (Charlie).

(7.2) Measurement-device-independent quantum secret sharing [16].
By exploiting the postselected GHZ state, one can also realize quantum secret sharing. We only consider the photon loss from quantum channels and detectors here. The secret key rate of using a single-photon source can be given by

$$R_{\rm QSS}=\frac{1}{4}\eta_d\eta^2. \tag{24}$$

Here, we have $\eta=\eta_d\times 10^{-\alpha L/10}$, $\eta_d=85\%$, $\alpha=0.167$ dB/km and assume that there is no loss of Alice's photon. The factor $1/4$ comes from only two of eight GHZ states that can be identified. L is the distance between Alice and Bob (Charlie).

(7.3) Round-robin quantum secret sharing [17].
The secret key rate per pulse of the round-robin quantum secret sharing with a twin-field for sending d coherent pulses each train in the case of the inside adversary can be written as

$$R_{\rm QSS}=\frac{1}{d}\{\hat{Q}[1-h(\hat{e}_{\rm p})]-Qfh(e_{\rm b})\}, \tag{25}$$

Supplementary Table S1. List of the experimental data used for one-time secure key generation.

	Alice-Bob (101 km)	Alice-Charlie (126 km)
n_μ^z	4616266	5225387
n_ν^z	258671	296285
n_ω^z	394425	437496
n_0^z	11189	14080
n_μ^x	1035046	1311484
n_ν^x	58107	78890
n_ω^x	87317	110362
n_0^x	3126	8168
m_ω^z	3446	9401

where we have $\hat{Q} = \min\{Q_A, Q_B\}$. Q_A and Q_B are the gains of Charlie's successful detection from Alice and Bob, respectively. The phase error rate is

$$\hat{e}_p = \frac{e_{\rm src}}{\hat{Q}} + \left(1 - \frac{e_{\rm src}}{\hat{Q}}\right)\frac{n_{\rm th}}{d-1}. \tag{26}$$

The average gain Q and bit error rate $e_{\rm b}$ of each train can be written as

$$Q = \frac{1}{2}[1 - (1 - dp_d)e^{-2\mu\eta}], \tag{27}$$

and

$$e_{\rm b} = \frac{e_d(1 - e^{-2\mu\eta}) + dp_d e^{-2\mu\eta}/2}{1 - (1 - dp_d)e^{-2\mu\eta}}, \tag{28}$$

where $\eta = \eta_d \times 10^{-\alpha L/10}$ is the efficiency between Alice and Bob (Charlie). In addition, we have the gains $Q_A = Q_B = Q/2$ due to the symmetry. Let n be the total photon number in a train of optical pulses with total intensity μ. Then, the probability of finding more than $n_{\rm th}$ photons in a train of optical pulses can be written as

$$\Pr(n > n_{\rm th}) = e_{\rm src} := 1 - \sum_{n=0}^{n_{\rm th}} \frac{e^{-\mu}\mu^n}{n!}, \tag{29}$$

where $n_{\rm th}$ is an integer constant chosen in this protocol. For the simulation, we set $p_d = 10^{-8}$, $f = 1.1$, $\eta_d = 85\%$, $\alpha = 0.167$ dB/km and $e_d = 2\%$. The intensity μ, the selected integer $n_{\rm th}$ and the number of optical pulses d are globally optimized.

(7.4) Single-qubit quantum secret sharing [18].
For the single-qubit quantum secret sharing protocol with a single photon source, the final key rate can be easily derived based on phase-error correction

$$R_{\rm QSS} = Q_1[1 - h(e_{\rm p}) - fh(e_{\rm b})], \tag{30}$$

where $e_{\rm p} = e_{\rm b} = p_d(1-2e_d)/Q_1 + e_d$ and $Q_1 = \mu e^{-\mu}(2p_d + \eta - 2p_d\eta)$. $\eta = \eta_d \times 10^{-\alpha\cdot 2L/10}$ is the total transmittance; $2L$ is the total distance. The intensity μ is globally optimized. We set $p_d = 10^{-8}$, $f = 1.1$, $\eta_d = 85\%$, $\alpha = 0.167$ dB/km and $e_d = 2\%$.

(7.5) Differential-phase-shift quantum secret sharing [19].
The final key rate for differential-phase-shift quantum secret sharing using a twin-field is bounded by

$$R_{\rm QSS} = Q_\mu[-(1-2\mu)\log_2(P_{\rm co}) - fh(E_\mu)], \tag{31}$$

where Q_μ is the gain of the whole system. $h(x) = -x\log_2(x) - (1-x)\log_2(1-x)$ is the Shannon entropy, and f is the error correction efficiency. $P_{\rm co}$ is the upper bound of collision probability when considering individual attacks,

which can be concluded as $P_{\mathrm{co}} = 1 - E_\mu^2 - (1-6E_\mu)^2/2$. The total gain and the total error rate with an intensity of μ are given by

$$\begin{aligned} Q_\mu &= 1-(1-2p_d)e^{-\mu\eta}, \\ E_\mu Q_\mu &= e_d Q_\mu + \left(\frac{1}{2}-e_d\right)2p_d e^{-\mu\eta}, \end{aligned} \tag{32}$$

where e_d is the misalignment error rate of detectors. $\eta = \eta_d \times 10^{-\alpha L/10}$ is the efficiency between Alice and Bob (Charlie), where η_d is the detection efficiency of Charlie's detectors. The intensity is globally optimized. We set $p_d = 10^{-8}$, $f = 1.16$, $\eta_d = 85\%$, $\alpha = 0.167$ dB/km and $e_d = 2\%$.

8. Experimental details

In the transmitting end, a master laser generates a repetition rate of 200 MHz and phase-randomized laser pulses 1.6 ns-wide at 1550.12 nm. To avoid using a phase modulator, we utilize two slave lasers to generate relative phases 0 and π by using the quantum properties of the beam splitter. An asymmetric interferometer with a 2 ns time delay divides each master pulse into two pairs of optical pulses with relative phases 0 and π. Then, the two pairs of optical pulses are injected into two slave lasers through the optical circulator. With the help of controlling the trigger electrical signal of two slave lasers, one generates a quantum signal only in the first time-bin or the second time-bin to constitute the Z basis, and one prepares a quantum signal both in two time-bins with a 0 or π phase difference to constitute the X basis. A 50 GHz bandwidth fiber Bragg grating is exploited to precompensate for pulse broadening and to remove extra spurious emission. The 2 ns-wide synchronization pulses with repetition rates of 100 kHz are transmitted via the quantum channel by using wavelength division multiplexed. The slave pulse width is 400 ps, which is much larger than the 10 ps timing resolution of the programmable delay chip, which means that the time consistency can be accurately calibrated. The spectral consistency is naturally satisfied through the laser seeding technique [20].

In the receiving end, a 30:70 biased beam splitter is used to perform passive basis detection after a wavelength division demultiplexer. A probability of 30% is measured in phase interference and the probability of 70% is used to receive in the time basis. A Faraday-Michelson interferometer realizes the phase measurement, where phase drift is compensated in real time by using the phase shifter. Two single photon detectors are used to measure the first time bin and the second time bin in the Z basis. Another two single photon detectors are used to measure the 0 and π phase difference in the X basis. The total insertion losses of the time and phase bases are 4.25 and 8 dB, respectively. The efficiency of single-photon detectors is 20% at a 160 dark count per second. To decrease the after-pulse probability, we set the dead times to 10 and 25 μs for the Bob-Alice and Charlie-Alice links, respectively.

A four-intensity decoy-state protocol [21–23] is adopted, where the intensities of the Z basis are set as $\mu = 0.35$ and $\nu = 0.15$, and the intensity of the X basis is $\omega = 0.3$. The intensity of the vacuum state is 0, which does not contain any basis information. The corresponding probabilities are $p_\mu = 0.78$, $p_\nu = 0.1$, $p_\omega = 0.08$ and $p_0 = 0.04$. Thereinto, the amplitude modulator generates two different intensities, and the intensity of ω is double that of ν since it has two pulses in the X basis.

The error correction and privacy amplification are carried out using a field-programmable gate array. Each time, privacy amplification will be performed after accumulating data approximately to the size of 4 Mb via approximately ten times of error correction, where the data size excludes the amount of information leaked in error correction. For the link of Alice-Bob with 101 km (Alice-Charlie with 126 km), one needs to accumulate approximately 153 (560) seconds of data for privacy amplification to extract a secure key. Here, we only list the experimental data of one set and calculation results related to privacy amplification, as shown in Tables S1 and S2.

To compare with the experimental results, we use the relevant experimental parameters to simulate the secure key rate, as shown in Table III. The length of the final key, which is $\varepsilon_{\mathrm{cor}}$-correct and $\varepsilon_{\mathrm{sec}}$-secret, can be given by [24, 25]

$$\ell = \underline{s}_0^{zz} + \underline{s}_1^{zz}\left[1 - h\left(\overline{\phi}_1^{zz}\right)\right] - \lambda_{\mathrm{EC}} - \log_2\frac{2}{\varepsilon_{\mathrm{cor}}} - 6\log_2\frac{22}{\varepsilon_{\mathrm{sec}}}, \tag{33}$$

where $h(x) := -x\log_2 x - (1-x)\log_2(1-x)$, and $\overline{x}$ ($\underline{x}$) denotes the upper (lower) bound of the observed value x. Using the decoy-state method for finite sample sizes, the expected numbers of vacuum events $\underline{s}_0^{zz^*}$ and single-photon events $\underline{s}_1^{zz^*}$ can be written as

$$\underline{s}_0^{zz^*} \geq (e^{-\mu}p_\mu + e^{-\nu}p_\nu)\frac{\underline{n}_0^{z^*}}{p_0}, \tag{34}$$

and

$$\underline{s}_1^{zz^*} \geq \frac{\mu^2 e^{-\mu}p_\mu + \mu\nu e^{-\nu}p_\nu}{\mu\nu - \nu^2} \times \left(e^\nu \frac{\underline{n}_\nu^{z^*}}{p_\nu} - \frac{\nu^2}{\mu^2}e^\mu\frac{\overline{n}_\mu^{z^*}}{p_\mu} - \frac{\mu^2-\nu^2}{\mu^2}\frac{\overline{n}_0^{z^*}}{p_0}\right), \tag{35}$$

Supplementary Table S2. List of the calculation results used for one-time secure key generation.

	Alice-Bob (101 km)	Alice-Charlie (126 km)
R_{QKD} (bps)	6021	470
Λ (s)	152.8	561
$\underline{s}_0^{zz}$	159429	203152
$\underline{s}_1^{zz}$	2756924	3158726
λ_{EC}	1225992	1659948
ℓ	920042	263482
$\overline{\phi}_1^{zz}$	4.83%	9.57%
ε_{sec}	10^{-10}	10^{-10}

Supplementary Table S3. Simulation parameters of the link between Alice-Bob (Charlie). e_d^z and e_d^x are the misalignment rates of the Z and X bases, and t_{dead} is the dead time of the detector.

Clock frequency	η_d	p_d	e_d^z	e_d^x	t_{dead}	ε_{cor}	f
2×10^8	20%	0.8×10^{-6}	2.3% (2.2%)	1.9% (2.4%)	10 (25) μs	10^{-15}	1.42

where $n_k^{z(x)}$ is the count of k ($k \in \{\mu,\ \nu,\ \omega\}$) intensity pulse measured in the $Z(X)$ basis, and x^* is the corresponding expected value of given observed value x. The upper and lower bounds can be acquired [26] by using the variant of the Chernoff bound, $\overline{x}^* = x + \beta + \sqrt{2\beta x + \beta^2}$ and $\underline{x}^* = x - \frac{\beta}{2} - \sqrt{2\beta x + \frac{\beta^2}{4}}$, where $\beta = \ln \frac{22}{\varepsilon_{\text{sec}}}$. The expected number of single-photon events $\underline{s}_1^{xx^*}$ in $\mathcal{X}_\omega$ can be given by [23]

$$\underline{s}_1^{xx^*} \geq \frac{\mu\omega e^{-\omega} p_\omega}{\mu\nu - \nu^2} \left(e^\nu \frac{\underline{n}_\nu^{x^*}}{p_\nu} - \frac{\nu^2}{\mu^2} e^\mu \frac{\overline{n}_\mu^{x^*}}{p_\mu} - \frac{\mu^2 - \nu^2}{\mu^2} \frac{\overline{n}_0^{x^*}}{p_0} \right). \tag{36}$$

In addition, the expected number of bit errors $\underline{t}_1^{xx^*}$ associated with the single-photon event in $\mathcal{X}_\omega$ is $\overline{t}_1^{xx} \leq m_\omega^x - \underline{t}_0^{xx}$, with $\underline{t}_0^{xx^*} = \frac{e^{-\omega} p_\omega}{2p_0} \underline{n}_0^{x^*}$. For a given expected value, the upper and lower bounds of the observed value can be given as $\overline{x} = x^* + \frac{\beta}{2} + \sqrt{2\beta x^* + \frac{\beta^2}{4}}$ and $\underline{x} = x^* - \sqrt{2\beta x^*}$, respectively. By using random sampling without replacement, the phase error rate in the Z basis is

$$\overline{\phi}_1^{zz} = \frac{\overline{t}_1^{xx}}{\underline{s}_1^{xx}} + \gamma^U \left(\underline{s}_1^{zz}, \underline{s}_1^{xx}, \frac{\overline{t}_1^{xx}}{\underline{s}_1^{xx}}, \frac{\varepsilon_{\text{sec}}}{22} \right), \tag{37}$$

where we have $\gamma^U(n,k,\lambda,\epsilon) = \frac{\frac{(1-2\lambda)AG}{n+k} + \sqrt{\frac{A^2G^2}{(n+k)^2} + 4\lambda(1-\lambda)G}}{2 + 2\frac{A^2G}{(n+k)^2}}$, with $A = \max\{n,k\}$ and $G = \frac{n+k}{nk} \ln \frac{n+k}{2\pi nk\lambda(1-\lambda)\epsilon^2}$.

Experimental demonstration the single-bit-type QDS of Ref. [27].
All the shared keys do not require error correction, and privacy amplification, i.e., n_μ, can be used for signature. For a one-bit message, suppose that $2L$ bits of keys are used. Then the security level of the signature can be bounded by [28]

$$\epsilon = max(P(honest\ abort), P(repudiation), P(forge)), \tag{38}$$

$$P(honest\ abort) = 2e^{-(s_a - \overline{E})^2 L}, \tag{39}$$

$$P(repudiation) = 2e^{-(s_v - s_a)^2 L}, \tag{40}$$

$$P(forge) = 2e^{-(p_e - s_v)^2 L}. \tag{41}$$

where we choose $s_a = \overline{E} + \frac{p_e - \overline{E}}{3}$, $s_v = \overline{E} + \frac{2(p_e - \overline{E})}{3}$, and $\overline{E}$ is the upper bound bit error rate of the signal state. p_e can be determined by

$$\underline{c}_0^{zz} + \underline{c}_1^{zz}[1 - h(\overline{\phi}_1^{zz})] = h(p_e) \tag{42}$$

where $\underline{c}_i^{zz} = \underline{s}_i^{zz}/n_\mu$.

Note that it is different between the Bob-Alice link and Charlie-Alice link. The parameters $\overline{E}$ and p_e should choose the maximal and minimum values, respectively. By using the experimental data, we can estimate $p_e = 7.06\%$, $s_v = 5.79\%$, $s_a = 4.52\%$ and $\overline{E} = 3.24\%$. Thus, to sign one bit, we need raw keys with $2L = 1.09 \times 10^6$ (4.66×10^5) bits, and the probability of honest abort, repudiation and forge are $P(honest\ abort) = P(repudiation) = P(forge) = \hat{\epsilon} = 10^{-38}$ (10^{-16}), respectively. When the length of the document is one megabit, the signature security bound $\hat{\epsilon} = 10^{-16}$ is $\epsilon = 1 - (1 - \hat{\epsilon}^{10^6}) \approx 10^6 \cdot \hat{\epsilon} = 10^{-32}$ (10^{-10}).

---

* hlyin@nju.edu.cn
† yfu@iphy.ac.cn
‡ zbchen@nju.edu.cn

[1] B. Schneier, *Secrets and lies: digital security in a networked world* (John Wiley & Sons, 2015).
[2] C. E. Shannon, Communication theory of secrecy systems, The Bell System Technical Journal **28**, 656 (1949).
[3] H. Krawczyk, Lfsr-based hashing and authentication, in *Annual International Cryptology Conference* (1994) pp. 129–139.
[4] A. J. Menezes, P. C. Van Oorschot, and S. A. Vanstone, *Handbook of applied cryptography* (CRC press, 2018).
[5] C.-H. F. Fung, X. Ma, and H. Chau, Practical issues in quantum-key-distribution postprocessing, Phys. Rev. A **81**, 012318 (2010).
[6] Y.-A. Chen, Q. Zhang, T.-Y. Chen, W.-Q. Cai, S.-K. Liao, J. Zhang, K. Chen, J. Yin, J.-G. Ren, Z. Chen, *et al.*, An integrated space-to-ground quantum communication network over 4,600 kilometres, Nature **589**, 214 (2021).
[7] M. O. Rabin, Probabilistic algorithms in finite fields, SIAM Journal on computing **9**, 273 (1980).
[8] J. Von Zur Gathen and J. Gerhard, *Modern computer algebra* (Cambridge university press, 2013).
[9] X.-B. Wang, Z.-W. Yu, and X.-L. Hu, Twin-field quantum key distribution with large misalignment error, Phys. Rev. A **98**, 062323 (2018).
[10] X. Ma, P. Zeng, and H. Zhou, Phase-matching quantum key distribution, Phys. Rev. X **8**, 031043 (2018).
[11] P. Zeng, W. Wu, and X. Ma, Symmetry-protected privacy: beating the rate-distance linear bound over a noisy channel, Phys. Rev. Appl. **13**, 064013 (2020).
[12] J. Lin, T. Upadhyaya, and N. Lütkenhaus, Asymptotic security analysis of discrete-modulated continuous-variable quantum key distribution, Phys. Rev. X **9**, 041064 (2019).
[13] J. Lodewyck, M. Bloch, R. García-Patrón, S. Fossier, E. Karpov, E. Diamanti, T. Debuisschert, N. J. Cerf, R. Tualle-Brouri, S. W. McLaughlin, and P. Grangier, Quantum key distribution over 25 km with an all-fiber continuous-variable system, Phys. Rev. A **76**, 042305 (2007).
[14] H.-K. Lo, M. Curty, and B. Qi, Measurement-device-independent quantum key distribution, Phys. Rev. Lett. **108**, 130503 (2012).
[15] M. Hillery, V. Bužek, and A. Berthiaume, Quantum secret sharing, Phys. Rev. A **59**, 1829 (1999).
[16] Y. Fu, H.-L. Yin, T.-Y. Chen, and Z.-B. Chen, Long-distance measurement-device-independent multiparty quantum communication, Phys. Rev. Lett. **114**, 090501 (2015).
[17] J. Gu, Y.-M. Xie, W.-B. Liu, Y. Fu, H.-L. Yin, and Z.-B. Chen, Secure quantum secret sharing without signal disturbance monitoring, Opt. Express **29**, 32244 (2021).
[18] C. Schmid, P. Trojek, M. Bourennane, C. Kurtsiefer, M. Żukowski, and H. Weinfurter, Experimental single qubit quantum secret sharing, Phys. Rev. Lett. **95**, 230505 (2005).
[19] J. Gu, X.-Y. Cao, H.-L. Yin, and Z.-B. Chen, Differential phase shift quantum secret sharing using a twin field, Opt. Express **29**, 9165 (2021).
[20] L. Comandar, M. Lucamarini, B. Fröhlich, J. Dynes, A. Sharpe, S.-B. Tam, Z. Yuan, R. Penty, and A. Shields, Quantum key distribution without detector vulnerabilities using optically seeded lasers, Nat. Photonics **10**, 312 (2016).
[21] X.-B. Wang, Beating the photon-number-splitting attack in practical quantum cryptography, Phys. Rev. Lett. **94**, 230503 (2005).
[22] H.-K. Lo, X. Ma, and K. Chen, Decoy state quantum key distribution, Phys. Rev. Lett. **94**, 230504 (2005).
[23] Z.-W. Yu, Y.-H. Zhou, and X.-B. Wang, Reexamination of decoy-state quantum key distribution with biased bases, Phys. Rev. A **93**, 032307 (2016).
[24] C. C. W. Lim, M. Curty, N. Walenta, F. Xu, and H. Zbinden, Concise security bounds for practical decoy-state quantum key distribution, Phys. Rev. A **89**, 022307 (2014).
[25] H.-L. Yin, P. Liu, W.-W. Dai, Z.-H. Ci, J. Gu, T. Gao, Q.-W. Wang, and Z.-Y. Shen, Experimental composable security decoy-state quantum key distribution using time-phase encoding, Opt. Express **28**, 29479 (2020).
[26] H.-L. Yin, M.-G. Zhou, J. Gu, Y.-M. Xie, Y.-S. Lu, and Z.-B. Chen, Tight security bounds for decoy-state quantum key distribution, Sci. Rep. **10**, 14312 (2020).

[27] R. Amiri, P. Wallden, A. Kent, and E. Andersson, Secure quantum signatures using insecure quantum channels, Phys. Rev. A **93**, 032325 (2016).

[28] X.-B. An, H. Zhang, C.-M. Zhang, W. Chen, S. Wang, Z.-Q. Yin, Q. Wang, D.-Y. He, P.-L. Hao, S.-F. Liu, *et al.*, Practical quantum digital signature with a gigahertz bb84 quantum key distribution system, Opt. Lett. **44**, 139 (2019).
